# Supplementary material for: Health Economic Evaluation of Lung Cancer Screening Using a Diagnostic Blood Test: The Early Detection of Cancer of the Lung Scotland (ECLS)
Source: Curr Oncol. 2024 Jun 18;31(6):3546–62. doi: 10.3390/curroncol31060261 (PMC11202544; doi:10.3390/curroncol31060261)
Supplement: Supplementary file 1 [file curroncol-31-00261-s001.zip › curroncol-3029056-supplementary.pdf]

Health economic evaluation of lung cancer screening using a diagnostic blood test: the Early detection of Cancer of the Lung Scotland (ECLS). Supplementary material.

Model parameters

Table S1: Description and estimation of model parameters

Parameter	Description	Estimation and assumptions
<u>Probabilities</u>		
Prevalence (p)	Prevalence of lung cancer (LC). It is the percentage of patients that have LC or will develop LC within the two years from randomisation, i.e. during the ECLS trial period.	Assumed based on ECLS trial protocol[1]. Changed in deterministic sensitivity analysis.
$p_{ES}=P(ES\|D+)$	Relative prevalence of early stage (ES) LC at randomisation point. If all patients with LC were screened during the ECLS trial with a perfect test (100% sensitivity), this would be the percentage of LCs that would be detected at ES.	Calibrated from the sensitivity of the EarlyCDT test and number of true positive cancers detected by the EarlyCDT: <br>$Number\ of\ ES\ cancers = Number\ of\ test\ positive\ ES/Sens\_Test_{ES}$ <br>$Number\ of\ LS\ cancers = Number\ of\ test\ positive\ LS/Sens\_Test_{LS}.$ <br>The parameter $P(ES\|D+)$ could be obtained as: $P(ES\|D+) = \frac{Number\ of\ ES\ cancers}{(Number\ of\ ES\ cancers + Number\ of\ LS\ cancers)}.$

Table S1: Description and estimation of model parameters

Parameter	Description	Estimation and assumptions
p_{LS}=*P(LS|D+)=1-* p_{ES}	Relative prevalence of late stage (LS) LC at randomisation point. If all patients with LC were screened during the ECLS trial with a perfect test (100% sensitivity), this would be the percentage of LCs that would be detected at LS.	See calibration of p_{ES} *= P(ES|D+).*
Test sensitivity_ES: $P(T^+\|ES)$	Sensitivity of the EarlyCDT®-Lung test for ES cancer.	Estimated in the ECLS study.[2]
Test sensitivity_LS: $P(T^+\|LS)$	Sensitivity of the EarlyCDT®-Lung test for LS cancer.	Estimated in the ECLS study. [2]
LDCT sensitivity: $P(LDCT^+\|D^+)$	Sensitivity of the LDCT scanning for any stage cancer.	Assumption.
LDCT specificity: $P(LDCT^-\|D^-)$	Specificity of the LDCT scanning for any stage cancer.	Assumption.
Opportunistic detection of ES cancer (Op_{ES})	Probability of oportunistic detection for an ES LC.	Ratio of the number of ES cases detected in the ECLS trial control arm over total number of ES cases expected.
Opportunistic detection of LS cancer (Op_{LS})	Probability of oportunistic detection for a LS LC.	Ratio of the number of LS cases detected in the ECLS trial control arm over total number of LS cases expected.
<u>Costs</u>		
Cost of EarlyCDT-Lung test	Cost of administering the EarlyCDT-Lung, including cost of the test kit and staff cost.	Assumed £106.5 in the base case analysis (15 minutes of nurse time included). Changed in deterministic sensitivity analysis.
Diagnostic costs	It comprises the cost incurred for investigations of LC (other than the cost of the EarlyCDT-Lung test).	The next estimations were used for each diagnostic pathway:<br>- TP, FP:<br>o Number of x-ray and LDCT scans as per ECLS trial screening protocol was the average found in the study for participants with a positive test result.

Table S1: Description and estimation of model parameters

Parameter	Description	Estimation and assumptions
		o Confirmatory diagnostic costs were assumed to be: an x-ray, a contrast LDCT, and the average of either a bronchoscopy or CT guided biopsy.<br>- TP (not LDCT screened)<br>o Confirmatory diagnostic costs as above for TP and FP.<br>- TN (LDCT screened), FN (LDCT screened)<br>o Number of x-ray and LDCT scans according to ECLS trial screening protocol. Estimated as above for TP and FP.<br>- TN, FN: no diagnostic costs assigned.<br><br>See unit costs of diagnostic procedures used in the analysis in Table A2 of this supplementary document.
Treatment costs	This is the costs of treatment that will be different for early stage and late stage cancer	Assumed as computed in the UK National Screening Committee (UK-NSC) report authored by the Exeter Test Group and Health Economics Group.[3] See table A7
Proportion of False Negative being treated after screening period	This parameter indicate what percentage of False Negative (i.e. lung cancer non-detected during the two-year ECLS intervention) will be detected before dying. These patients are assumed to be able to receive treatment and incurr in related costs.	Based on *Opportunistic detection of LS cancer (Op_{LS})* and survival function calibrated for LS cancers. See details in this supplementary material "Proportion of False Negative detected after the screening intervention"
Proportion of ES LC being treated after recurrence	Proportion of lung cancers, detected at early stage, that recur in a late stage and receive treatment and incur in related costs.	Based on CRUK report "Saving lives, averting costs"[4]
<u>Outcomes</u>		

Table S1: Description and estimation of model parameters

Parameter	Description	Estimation and assumptions
Lead time	This parameter refers to the average time passed between randomisation point and cancer detecction.	The next estimations were used for:<br>- LC detected at ES: average lead time for LC cases with a positive test result in the ECLS trial intervention arm.<br>- LC detected at LS: average lead time for LC cases in the control arm and LC cases with a negative test result in the intervention arm.
LE_ES	Life expectancy for ES LC.	The estimation of life expectancy for ES LC has three steps: 1) Estimation of Weibull survival function using digitised K-M curves from the International Association for the Study of LC (IASLC) staging project *[5]*; 2) Calibration of scale parameter λ using survival data from the UK; 3) Computation of Life expectancy for lung cancer at early stages by integrating the Weibull function using parameters estimated/calibrated in 1) and 2). See details in this supplementary material "Estimation of life expectancy".
LE_LS (non-treated)	Life expectancy for LS LC (non-treated).	The estimation of life expectancy for LS LC (non-treated) has three steps: 1) Estimation of Weibull survival function using digitised K-M curves from the International Association for the Study of LC (IASLC) staging project *[5]*; 2) Calibration of scale parameter λ using survival data from the UK; 3) Computation of Life expectancy for lung cancer at late stages by integrating the Weibull function using parameters estimated/calibrated in 1) and 2). See details in this supplementary material "Estimation of life expectancy".
LE_LS (treated)	Life expectancy for LS LC (immunotherapy-treated).	The estimation of life expectancy for LS LC (immunotherapy-treated) has four steps: 1) Estimation of Weibull survival function using digitised K-M curves from the International

Table S1: Description and estimation of model parameters

Parameter	Description	Estimation and assumptions
		Association for the Study of LC (IASLC) staging project *[5]*; 2) Calibration of scale parameter λ using survival data from the UK; 3) Adjusting mortality hazard by applying HR estimated in the literature for immunotherapy vs. chemotherapy[6]; 4) Computation of Life expectancy for lung cancer at early and late stages by integrating the Weibull function using parameters estimated/calibrated in 1), 2) and 3). See details in this supplementary material "Estimation of life expectancy".
HU_NLC	Health utility for no LC patients	Estimated from ECLS data for participants who did not develop LC.
HU_LC_change	Decrement utility for LC in general	Estimated from ECLS data for participants who developed LC. Assumed to be a general utility decrement that applies at the lowest stage of lung cancer. Additional decrements were applied to ES and LS LCs.
HU_ES_change	Additional decrement utility for ES LC	Health utilities estimated in Grutters et al. (2010) were used to apply utility decrements for early and late stage of LC. [7] See in this supplementary material "Health utilities".
HU_LS_change	Additional decrement utility for LS LC	Health utilities estimated in Grutters et al. (2010) were used to apply utility decrements for early and late stage of LC. [7] See in this supplementary material "Health utilities".

Unit costs

Table S2: Unit costs (2021-22 pounds sterling)

Resource use	Unit cost (£)	Source	Details
Blood test kit	95.00	Oncimmune	$124 per kit
Test administration	11.5	PSSRU 2021/22	15 minutes of nurse time (GP surgery) (£46 per hour)
LDCT scan one area	95.5	NHS reference costs 2017/18 and updated to 2022 using NHS Cost Inflation Index for prices	CT of one area, no contrast RD20A in diagnostic imaging tab, outpatient
LDCT scan two areas	113.5	NHS reference costs 2017/18 and updated to 2022 using NHS Cost Inflation Index for prices	CT scan of two areas with contrast (chest and abdomen) RD24Z, diagnostic imaging tab, outpatient
X-ray	64.5	ISD costs 2017/18 and updated to 2022 using NHS Cost Inflation Index for prices	Other radiology R120X
Bronchoscopy	954.73	NHS reference costs 2021/22	DZ69A respiratory medicine service diag bronch OP procedures
CT guided biopsy	1547.73	NHS reference costs 2021/22	DZ63C daycase major thoracic procedure 19+ CC score 0-2

Estimation of life expectancy at diagnosis

A Weibull model was used to compute life expectancy from diagnosis for each LC at stage i:

$$\int_0^\infty S_i(t) = \int_0^\infty \exp\left(-\lambda_i t^p\right). \quad (1)$$

Where $S_i(t)$ is the survival function and t=0 is the moment of diagnosis. Parameters p and λ_i were first estimated using digitized data from K-M curves published by the IASLC staging project.*[5]* Then, parameter λ_i was calibrated for each stage I, II, III and IV using five-year survival figures from England data:

$$\lambda_i = \frac{-\log\left(S_i(5)\right)}{5^p}. \quad (2)$$

Where log is the natural logarithm and $S_i(5)$ is the five-year survival figure using data available from England.

The estimation of life expectancy has three steps: 1) Estimation of Weibull survival function using digitised K-M curves from the International Association for the Study of LC (IASLC) staging project*[5]*; 2) Calibration of scale parameter λ using survival data from the UK; 3) Computation of Life expectancy for each stage by integrating the Weibull function using parameters estimated/calibrated in 1) and 2).

The estimation of life expectancy for LS LC (immunotherapy-treated) is computed seemingly but applying a HR, estimated in the literature for immunotherapy vs. chemotherapy, to the scale parameters λ. [6]

<u>Estimation of Weibull survival function</u>

The IASLC staging project presented data for 17,509 observations representing patients in different LC stages (see figure 2A in *[5]*). Data covers a period of 72 months from diagnosis. Given that no information is shown on censoring during this period, all censoring was assumed to happen at the end of the period.

Table S3. Weibull survival function estimation

	Coef.	S.E.
Log(λ):		
Stage IB	0.617	0.041
Stage IIA	1.040	0.050
Stage IIB	1.198	0.053
Stage IIIA	1.520	0.033
Stage IIIB	2.065	0.034
Stage IV	2.654	0.044
Cons (Stage IA)	-2.875	0.029
Log(p)	-0.143	0.009
N	17,509	

Note: digitized data from the IASLC staging project used

Calibration of scale parameter λ to UK data

Data from England NHS-Digital publication "*Cancer Survival in England, cancers diagnosed 2015 to 2019, followed up to 2020*" is shown in Table A4.[8]

Table S4. 5-year age-standardised survival ($S_i(5)$) for LC. England NHS-Digital

Stage 1	Stage 2	Stage 3	Stage 4
0.609	0.387	0.147	0.039

The average of the scale parameter λ_i for each stage i is calibrated to meet five-year survival figures. A standard error for the scale parameter is applied for each scale parameter using estimations in Table A3.

Table S5. Calibration of Weibull scale parameter (λ)

	Log(λ)			
	Stage 1	Stage 2	Stage 3	Stage 4
Average	-1.959	-1.322	-0.667	-0.131
S.E.	0.029	0.045	0.024	0.035

Note. Standard errors taken from estimations of λ for each stage in Table A3.

Also, the HR used to adjust the mortality hazard for immunotherapy-treated LS LC is 0.62 (95% CI, 0.48 to 0.81).[6] A lognormal distribution, $logHR{\sim}N(-0.478, 0.135)$, has been used to account for uncertainty in the HR, where the standard deviation 0.135 has been calibrated to be consistent with the 95% confidence interval in the HR.

Computation of Life expectancy

Life expectancy is computed by integrating the Weibull survival function using the estimated shape parameter and calibrated scale parameter (adjusted for immunotherapy treatment as appropriate). The results are shown in Table A6 below. The average of life expectancy for stage 1 and stage 2 are applied to cancers detected at early stage in our model. Late-stage cancers are assigned the average for stage 3 and stage 4.

Table S6. Life expectancy for each LC stage

	Stage 1	Stage 2	Stage 3 (non-treated)	Stage 4 (non-treated)	Stage 3 (immun-treated)	Stage 4 (immun-treated)
Average	12.047	5.703	2.538	1.384	4.405	2.401
S.E.	0.430	0.271	0.0758	0.0571	0.697	0.396

Treatment costs

The CRUK report "Saving lives, averting costs" presented treatment costs for LC patients according to their disease stage. [4] Early-stage LC treatment costs were computed as the average of stage I and II.

Table S7: Treatment costs (£) at diagnosis from the UK National Screening Committee (UK-NSC) report

Type of treatment	Lung cancer stage			
	I	II	III	IV
Surgery	2982	2895	847	549
Radio Therapy (RT)	909	791	472	730
Chemo-rad			1817	
Chemo Therapy (CT)				782
CT doublet		587	105	393
Immunotherapy			8426	2640
Chemo-immunotherapy			3585	6692
TKI			1334	3772
Follow-up	271	217	162	108
Total	4162	4490	16748	15666

Late-stage lung cancers were assigned the average of treatment cost for stage III and IV. Early-stage lung cancers were assigned the average of treatment costs for stage I and II.

Proportion of False Negative detected after the screening intervention.

In the model, False Negative patients did not receive a correct diagnosis during the period of the ECLS intervention and therefore were assumed to have been detected later at an advanced stage. But not all of them will be diagnosed before dying. It was assumed that they would be detected according to opportunistic detection rate for LS LC. Using the opportunistic detection rate during the two years of the ECLS intervention (0.7208), we can compute the one-year probability of detection as $opp = 1 - (1 - 0.7208)^{1/2}$. The proportion of False Negative patients detected before dying can be computed as:

$$\sum_{t=2}^{t=T} FN_{\mathrm{det}(t)} = \sum_{t=2}^{t=T} \left(opp \times \left[S(t) \left(1 - \sum_{i=2}^{t} FN_{\mathrm{det}(i)} \right) \right] \right),$$

where *t* is the index for year since end of screening intervention at year 2, T is the year where all the False Negative are dead, $FN_{\mathrm{det}(t)}$ are the False Negative patients detected in year t, and $S(t)$ is the proportion of False Negative that survives to year t. Using the Weibull

survival function for LS LC we obtain $\sum_{t=2}^{t=T} FN_{\text{det}(t)} = 0.3885$. The average year of detection, when treatment is expected to happen for these patients, is t=3.

Health utilities

Health utilities estimated in Grutters et al. (2010) were used to apply utility decrements different stages of LC. [7] Based on these figures additional utility decrements in the model were 0.03 and 0.07 for early and late stage LC.

Table S8. Utility decrements based on Grutters et al. (2010)

	Original table			Model parameters	
Stage	N	Mean	Sd	Utility decrements w.r.t. stage I	S.E.
I	105	0.77	0.26	0	0.025
II	39	0.74	0.22	-0.03	0.035
III	99	0.7	0.29	-0.07	0.029
IV	2	0.86	0.19	-0.07	0.029

Note: Given lack of data, model parameters for stage IV are assumed the same as for stage III.

Cohort counts for each model pathway (baseline results). Cohort of 1,000 patients

Table S9. Cohort counts for each model pathway. Baseline results. 1,000 patients

	Endpoint (diagnosis pathway and cancer stage at diagnosis)										
Strategy	FP (NLC)	TN LDCT-screened (NLC)	TN (NLC)	TP (ES)	FN LDCT-screened (LS)	TP NotLDCT-screened (ES)	FN (LS)	TP (LS)	FN LDCT-screened (LS)	TP NotLDCT-screened (LS)	FN (LS)
ECLS intervention	0	94.2	886	4.29	0	1.48	2.44	2.14	0	6.95	2.69
Comparator 1: No screening	0	0	980	0	0	3.1	5.11	0	0	8.5	3.29
Comparator 2: LDCT screening	0	980	0	8.21	0	0	0	11.8	0	0	0

TN – true negative; TP – true positive; FN – false negative; FP – false positive; LDCT – low-dose computed tomography; LC – lung cancer; NLC – no lung cancer; ES – early stage; LS – late-stage

Table S10. Deterministic sensitivity analysis

Parameter assumption	ECLS intervention	Comp. 1: No screening	Comp. 2: LDCT screening
EarlyCDT cost (£201.5)			
Costs (£)	509,102	232,421	796,016
QALYs	8,570.4	8,559.7	8,581.4
NMB (λ=£20,000)	170,899,000	170,961,000	170,832,000
NMB (λ=£30,000)	256,603,000	256,557,000	256,645,000
ΔCosts (£)		276,681	-286,915
ΔQALYs		10.7	-11.0
ΔNMB (λ=£20,000)		-61,821	67,095
ΔNMB (λ=£30,000)		45,609	-42,816
EarlyCDT cost (£59)			
Costs (£)	366,602	232,421	796,016
QALYs	8,570.4	8,559.7	8,581.4
NMB (λ=£20,000)	171,041,000	170,961,000	170,832,000
NMB (λ=£30,000)	256,745,000	256,557,000	256,645,000
ΔCosts (£)		134,181	-429,415
ΔQALYs		10.7	-11.0
ΔNMB (λ=£20,000)		80,679	209,595
ΔNMB (λ=£30,000)		188,109	99,685
Lead time (0.4443)			
Costs (£)	414,102	232,421	796,016
QALYs	8,565.2	8,553.4	8,577.1
NMB (λ=£20,000)	170,889,000	170,836,000	170,746,000
NMB (λ=£30,000)	256,541,000	256,370,000	256,517,000
ΔCosts (£)		181,681	-381,915
ΔQALYs		11.7	-11.9
ΔNMB (λ=£20,000)		53,219	143,294
ΔNMB (λ=£30,000)		170,669	23,985
Lead time (2)			
Costs (£)	414,102	232,421	796,016
QALYs	8,578.4	8,569.2	8,588.0
NMB (λ=£20,000)	171,154,000	171,152,000	170,963,000
NMB (λ=£30,000)	256,938,000	256,844,000	256,842,000

Parameter assumption	ECLS intervention	Comp. 1: No screening	Comp. 2: LDCT screening
ΔCosts (£)		181,681	-381,915
ΔQALYs		9.2	-9.5
ΔNMB (λ=£20,000)		2,359	191,074
ΔNMB (λ=£30,000)		94,379	95,655
Opportunistic detection of ES cancer (0)			
Costs (£)	403,428	210,103	796,016
QALYs	8,564.6	8,547.5	8,581.4
NMB (λ=£20,000)	170,888,000	170,740,000	170,832,000
NMB (λ=£30,000)	256,534,000	256,215,000	256,645,000
ΔCosts (£)		193,325	-392,588
ΔQALYs		17.1	-16.8
ΔNMB (λ=£20,000)		148,175	56,688
ΔNMB (λ=£30,000)		318,925	-111,262
Opportunistic detection of ES cancer (1)			
Costs (£)	431,673	269,162	796,016
QALYs	8,580.0	8,579.6	8,581.4
NMB (λ=£20,000)	171,167,000	171,323,000	170,832,000
NMB (λ=£30,000)	256,967,000	257,120,000	256,645,000
ΔCosts (£)		162,512	-364,343
ΔQALYs		0.3	-1.4
ΔNMB (λ=£20,000)		-156,132	335,623
ΔNMB (λ=£30,000)		-152,942	321,263
Opportunistic detection of LS cancer (0)			
Costs (£)	335,141	135,913	796,016
QALYs	8,566.7	8,555.1	8,581.4
NMB (λ=£20,000)	170,999,000	170,966,000	170,832,000
NMB (λ=£30,000)	256,665,000	256,518,000	256,645,000
ΔCosts (£)		199,228	-460,875
ΔQALYs		11.6	-14.7
ΔNMB (λ=£20,000)		32,112	166,895

Parameter assumption	ECLS intervention	Comp. 1: No screening	Comp. 2: LDCT screening
ΔNMB (λ=£30,000)		147,782	19,905
Opportunistic detection of LS cancer (1)			
Costs (£)	444,684	269,799	796,016
QALYs	8,571.8	8,561.4	8,581.4
NMB (λ=£20,000)	170,992,000	170,958,000	170,832,000
NMB (λ=£30,000)	256,710,000	256,572,000	256,645,000
ΔCosts (£)		174,885	-351,332
ΔQALYs		10.4	-9.6
ΔNMB (λ=£20,000)		33,595	160,232
ΔNMB (λ=£30,000)		137,835	64,682
*Prob. of treatment undetected LC (*0.19*)*			
Costs (£)	399,194	208,039	796,016
QALYs	8,569.5	8,558.2	8,581.4
NMB (λ=£20,000)	170,991,000	170,955,000	170,832,000
NMB (λ=£30,000)	256,685,000	256,537,000	256,645,000
ΔCosts (£)		191,156	-396,822
ΔQALYs		11.3	-11.9
ΔNMB (λ=£20,000)		35,204	158,882
ΔNMB (λ=£30,000)		148,384	39,912
*Prob. of treatment undetected LC (*0.76*)*			
Costs (£)	441,990	278,034	796,016
QALYs	8,572.1	8,562.4	8,581.4
NMB (λ=£20,000)	170,999,000	170,970,000	170,832,000
NMB (λ=£30,000)	256,720,000	256,594,000	256,645,000
ΔCosts (£)		163,956	-354,027
ΔQALYs		9.7	-9.3
ΔNMB (λ=£20,000)		29,504	167,847
ΔNMB (λ=£30,000)		126,234	74,757
Discount rate (6%)			
Costs (£)	405,102	223,735	780,617
QALYs	7,615.0	7,605.7	7,624.6

Parameter assumption	ECLS intervention	Comp. 1: No screening	Comp. 2: LDCT screening
NMB (λ=£20,000)	151,895,000	151,890,000	151,712,000
NMB (λ=£30,000)	228,045,000	227,946,000	227,958,000
ΔCosts (£)		181,367	-375,515
ΔQALYs		9.3	-9.6
ΔNMB (λ=£20,000)		5,393	183,275
ΔNMB (λ=£30,000)		98,773	87,155
Discount rate (0%)			
Costs (£)	427,898	245,745	819,239
QALYs	10,284.4	10,271.2	10,297.8
NMB (λ=£20,000)	205,261,000	205,179,000	205,137,000
NMB (λ=£30,000)	308,105,000	307,891,000	308,115,000
ΔCosts (£)		182,153	-391,341
ΔQALYs		13.2	-13.4
ΔNMB (λ=£20,000)		81,847	123,341
ΔNMB (λ=£30,000)		213,847	-10,659
Prevalence (1%)			
Costs (£)	283,707	116,210	641,390
QALYs	8,620.1	8,614.7	8,625.6
NMB (λ=£20,000)	172,118,000	172,178,000	171,870,000
NMB (λ=£30,000)	258,319,000	258,325,000	258,126,000
ΔCosts (£)		167,497	-357,683
ΔQALYs		5.4	-5.5
ΔNMB (λ=£20,000)		-60,077	247,763
ΔNMB (λ=£30,000)		-6,367	192,803
Prevalence (4%)			
Costs (£)	674,891	464,841	1,105,270
QALYs	8,471.0	8,449.5	8,493.0
NMB (λ=£20,000)	168,745,000	168,526,000	168,755,000
NMB (λ=£30,000)	253,456,000	253,021,000	253,685,000
ΔCosts (£)		210,050	-430,378
ΔQALYs		21.5	-22.0
ΔNMB (λ=£20,000)		219,670	-9,282

Parameter assumption	ECLS intervention	Comp. 1: No screening	Comp. 2: LDCT screening
ΔNMB (λ=£30,000)		434,530	-229,112
Relative prevalence ES (0.25)			
Costs (£)	425,855	250,008	810,712
QALYs	8,562.9	8,556.1	8,570.5
NMB (λ=£20,000)	170,832,000	170,873,000	170,600,000
NMB (λ=£30,000)	256,461,000	256,434,000	256,305,000
ΔCosts (£)		175,847	-384,857
ΔQALYs		6.8	-7.6
ΔNMB (λ=£20,000)		-40,827	231,977
ΔNMB (λ=£30,000)		26,683	155,537
Relative prevalence ES (0.75)			
Costs (£)	389,289	195,292	764,991
QALYs	8,586.2	8,567.1	8,604.3
NMB (λ=£20,000)	171,335,000	171,146,000	171,321,000
NMB (λ=£30,000)	257,198,000	256,817,000	257,364,000
ΔCosts (£)		193,997	-375,702
ΔQALYs		19.2	-18.1
ΔNMB (λ=£20,000)		189,403	14,542
ΔNMB (λ=£30,000)		381,103	-166,038
*Prob. recurrence (*0.25*)*			
Costs (£)	393,014	221,077	765,997
QALYs	8,569.3	8,559.1	8,579.8
NMB (λ=£20,000)	170,993,000	170,960,000	170,830,000
NMB (λ=£30,000)	256,686,000	256,551,000	256,629,000
ΔCosts (£)		171,938	-372,983
ΔQALYs		10.2	-10.5
ΔNMB (λ=£20,000)		32,762	162,463
ΔNMB (λ=£30,000)		135,112	57,203
*Prob. recurrence (*0.75*)*			
Costs (£)	435,189	243,764	826,035
QALYs	8,571.5	8,560.2	8,582.9
NMB (λ=£20,000)	170,994,000	170,961,000	170,832,000

Parameter assumption	ECLS intervention	Comp. 1: No screening	Comp. 2: LDCT screening
NMB (λ=£30,000)	256,709,000	256,563,000	256,661,000
ΔCosts (£)		191,425	-390,846
ΔQALYs		11.2	-11.4
ΔNMB (λ=£20,000)		33,295	161,986
ΔNMB (λ=£30,000)		145,655	47,556
Test sensitivity_ES (0.25*)*			
Costs (£)	403,257	232,421	796,016
QALYs	8,565.0	8,559.7	8,581.4
NMB (λ=£20,000)	170,896,000	170,961,000	170,832,000
NMB (λ=£30,000)	256,546,000	256,557,000	256,645,000
ΔCosts (£)		170,836	-392,759
ΔQALYs		5.3	-16.4
ΔNMB (λ=£20,000)		-64,556	64,359
ΔNMB (λ=£30,000)		-11,416	-99,841
Test sensitivity_ES (0.75*)*			
Costs (£)	423,211	232,421	796,016
QALYs	8,575.0	8,559.7	8,581.4
NMB (λ=£20,000)	171,076,000	170,961,000	170,832,000
NMB (λ=£30,000)	256,825,000	256,557,000	256,645,000
ΔCosts (£)		190,791	-372,805
ΔQALYs		15.3	-6.4
ΔNMB (λ=£20,000)		115,269	244,185
ΔNMB (λ=£30,000)		268,299	179,875
Test sensitivity_LS (0.09*)*			
Costs (£)	409,817	232,421	796,016
QALYs	8,570.2	8,559.7	8,581.4
NMB (λ=£20,000)	170,995,000	170,961,000	170,832,000
NMB (λ=£30,000)	256,697,000	256,557,000	256,645,000
ΔCosts (£)		177,397	-386,199
ΔQALYs		10.6	-11.2
ΔNMB (λ=£20,000)		34,243	163,159
ΔNMB (λ=£30,000)		140,063	51,639

Parameter assumption	ECLS intervention	Comp. 1: No screening	Comp. 2: LDCT screening
*Test sensitivity_LS (*0.36*)*			
Costs (£)	422,415	232,421	796,016
QALYs	8,570.7	8,559.7	8,581.4
NMB (λ=£20,000)	170,992,000	170,961,000	170,832,000
NMB (λ=£30,000)	256,699,000	256,557,000	256,645,000
ΔCosts (£)		189,995	-373,601
ΔQALYs		11.1	-10.7
ΔNMB (λ=£20,000)		31,125	160,041
ΔNMB (λ=£30,000)		141,685	53,261
*Test specificity (*0.5*)*			
Costs (£)	606,740	232,421	796,016
QALYs	8,570.4	8,559.7	8,581.4
NMB (λ=£20,000)	170,801,000	170,961,000	170,832,000
NMB (λ=£30,000)	256,505,000	256,557,000	256,645,000
ΔCosts (£)		374,319	-189,276
ΔQALYs		10.7	-11.0
ΔNMB (λ=£20,000)		-159,459	-30,544
ΔNMB (λ=£30,000)		-52,029	-140,454
Test specificity (1)			
Costs (£)	368,226	232,421	796,016
QALYs	8,570.4	8,559.7	8,581.4
NMB (λ=£20,000)	171,040,000	170,961,000	170,832,000
NMB (λ=£30,000)	256,744,000	256,557,000	256,645,000
ΔCosts (£)		135,805	-427,790
ΔQALYs		10.7	-11.0
ΔNMB (λ=£20,000)		79,055	207,970
ΔNMB (λ=£30,000)		186,485	98,060
*Health utility for no LC (*0.5*)*			
Costs (£)	414,102	232,421	796,016
QALYs	4,951.9	4,946.4	4,957.5
NMB (λ=£20,000)	98,623,300	98,694,900	98,352,900
NMB (λ=£30,000)	148,142,000	148,159,000	147,927,000

Parameter assumption	ECLS intervention	Comp. 1: No screening	Comp. 2: LDCT screening
ΔCosts (£)		181,681	-381,915
ΔQALYs		5.5	-5.6
ΔNMB (λ=£20,000)		-71,621	270,355
ΔNMB (λ=£30,000)		-16,591	214,575
Health utility for no LC (1)			
Costs (£)	414,102	232,421	796,016
QALYs	9,920.9	9,908.2	9,933.9
NMB (λ=£20,000)	198,004,000	197,932,000	197,882,000
NMB (λ=£30,000)	297,213,000	297,014,000	297,222,000
ΔCosts (£)		181,681	-381,915
ΔQALYs		12.7	-13.0
ΔNMB (λ=£20,000)		72,299	121,695
ΔNMB (λ=£30,000)		199,289	-8,416
Health utility for no LC (1)			
Costs (£)	409,817	232,421	796,016
QALYs	8,570.2	8,559.7	8,581.4
NMB (λ=£20,000)	170,995,000	170,961,000	170,832,000
NMB (λ=£30,000)	256,697,000	256,557,000	256,645,000
ΔCosts (£)		177,397	-386,199
ΔQALYs		10.6	-11.2
ΔNMB (λ=£20,000)		34,243	163,159
ΔNMB (λ=£30,000)		140,063	51,639
Health utility for no LC (1)			
Costs (£)	422,415	232,421	796,016
QALYs	8,570.7	8,559.7	8,581.4
NMB (λ=£20,000)	170,992,000	170,961,000	170,832,000
NMB (λ=£30,000)	256,699,000	256,557,000	256,645,000
ΔCosts (£)		189,995	-373,601
ΔQALYs		11.1	-10.7
ΔNMB (λ=£20,000)		31,125	160,041
ΔNMB (λ=£30,000)		141,685	53,261
Health utility for no LC (1)			
Costs (£)	440,808	232,421	1,073,720

Parameter assumption	ECLS intervention	Comp. 1: No screening	Comp. 2: LDCT screening
QALYs	8,570.4	8,559.7	8,581.4
NMB (λ=£20,000)	170,967,000	170,961,000	170,554,000
NMB (λ=£30,000)	256,671,000	256,557,000	256,368,000
ΔCosts (£)		208,387	-632,907
ΔQALYs		10.7	-11.0
ΔNMB (λ=£20,000)		6,473	413,087
ΔNMB (λ=£30,000)		113,903	303,177
Health utility for no LC (1)			
Costs (£)	424,784	232,421	907,096
QALYs	8,570.4	8,559.7	8,581.4
NMB (λ=£20,000)	170,983,000	170,961,000	170,721,000
NMB (λ=£30,000)	256,687,000	256,557,000	256,534,000
ΔCosts (£)		192,364	-482,312
ΔQALYs		10.7	-11.0
ΔNMB (λ=£20,000)		22,497	262,492
ΔNMB (λ=£30,000)		129,927	152,582

REFERENCES

1. Sullivan, F.M., et al., *Detection in blood of autoantibodies to tumour antigens as a case-finding method in lung cancer using the EarlyCDT((R))-Lung Test (ECLS): study protocol for a randomized controlled trial.* Bmc Cancer, 2017. **17**.
2. Sullivan, F.M., et al., *Earlier diagnosis of lung cancer in a randomised trial of an autoantibody blood test followed by imaging.* European Respiratory Journal, 2021. **57**(1).
3. Exeter-Test-Group-and-Health-Economics-Group, *Interim report on the cost-effectiveness of low dose computed tomography (LDCT) screening for lung cancer in high risk individuals*. 2022.
4. Cancer Research UK. *Saving lives, averting costs*. 2019 16.10.19].
5. Goldstraw, P., et al., *The IASLC lung cancer staging project: proposals for revision of the TNM stage groupings in the forthcoming (eighth) edition of the TNM classification for lung cancer.* Journal of Thoracic Oncology, 2016. **11**(1): p. 39-51.
6. Reck, M., et al., *Five-Year Outcomes With Pembrolizumab Versus Chemotherapy for Metastatic Non–Small-Cell Lung Cancer With PD-L1 Tumor Proportion Score ≥ 50%.* Journal of Clinical Oncology, 2021. **39**(21): p. 2339-2349.
7. Grutters, J.P., et al., *Health-related quality of life in patients surviving non-small cell lung cancer.* Thorax, 2010. **65**(10): p. 903-907.
8. NHS-digital, *Cancer Survival in England, cancers diagnosed 2015 to 2019, followed up to 2020*, in *National statistics*. 2022.
